# Exogenous Feeding of Fructose and Phenylalanine Further Improves Betulin Production in Suspended *Betula platyphylla* Cells under Nitric Oxide Treatment

**DOI:** 10.3390/molecules22071035

**Published:** 2017-06-30

**Authors:** Guizhi Fan, Tingting Nie, Jin Sheng Fan, Yaguang Zhan

**Affiliations:** 1 College of Life Science, Northeast Forestry University, Harbin 150040, China; gzf325@126.com (G.F.); 17745188284@163.com (T.N.); 2 College of Resources and Environmental Science, Heilongjiang University, Harbin 150040, China; firstjs@163.com

**Keywords:** *Betula platyphylla*, betulin, feeding strategy, metabolic profiling, nitric oxide

## Abstract

The aim of this study was to assay by NMR the metabolites which contribute to betulin production. 8-day-old suspended birch (*Betula platyphylla*) cells were treated by sodium nitroprusside (SNP) treatment, an NO donor, and 2-(4-carboxyphenyl)-4,4,5,5-tetramethyl-imidazoline-1-oxyl-3-oxide (cPTIO), an NO-specific scavenger. The results showed that betulin production was increased by five times after SNP treatment, similar with that of the control under cPTIO treatment. Forty one metabolites were detected after SNP treatment or cPTIO treatment. Among them, 10 were found to significantly contribute to the differences observed between controls and treated cell culture samples. To validate the contribution of the above 10 metabolites to betulin production, myo-inositol, fructose and phenylalanine based on correlation analysis between the content of 12 metabolites and betulin were used to feed birch suspension cell cultures under SNP treatment. Exogenous feeding of fructose or phenylalanine further enhanced the betulin production under SNP treatment, but myo-inositol had the opposite result.

## 1. Introduction

In vitro experimentation on plant tissue cultures gives a wide range of secondary metabolites that are used as therapeutics, flavors, fragrances, colors, and food additives [1,2]. For increasing the entire biomass and production of high amounts of secondary metabolites in plant cell cultures, the use of biotic and abiotic elicitors has been recognised as one of the most effective strategies [3,4]. Some such pharmaceutical secondary metabolites, the triterpenoids betulin and oleanolic acid, can be extracted from the bark of *Betula platyphylla* Suk. Triterpenoids are excellent drug candidates, with antiviral, antibacterial, antitumor, and anti-AIDS activity [5,6,7]. In our previous study, betulin was found in *B. platyphylla* cell cultures, and nitric oxide (NO) was shown to be an effective elicitor to stimulate triterpenoid synthesis in *Betula platyphylla* cell cultures [8,9].

Nitric oxide (NO) is a small gaseous radical, defined as a multi-purpose molecule, which is present throughout the plant life cycle [10]. It is involved in diverse physiological processes such as stomatal closure, as well as pathophysiological processes such as biotic and abiotic stress responses [11]. The biosynthesis of NO and plant secondary metabolites was reported to protect plants from attack by insect, herbivores, and pathogens, or to survive under abiotic stresses, and NO also plays important roles in the accumulation of secondary metabolites in plants [12], so a better understanding of the role of NO in the biosynthesis of such secondary metabolites is very important for optimizing the commercial production of those pharmaceutically significant secondary metabolites. However, holistic approaches to detect a wide range of metabolite changes due to the effect of NO have not yet been performed for betulin synthesis in *B. platyphylla* cell cultures.

Metabonomics aims to compare the relative differences between biological samples based on their metabolite profiles. It can provide a snapshot of the entire physiology of an organism [13]. Metabonomics, therefore, may be a good method to get a ‘holistic view’ of the actual responses of plants to NO elicitor. So, in this study, Proton Nuclear Magnetic Resonance (^1^H-NMR)-based metabonomics was employed to characterize the metabolic profile of the intracellular metabolites change in response to NO elicitor. The objective of the work reported here was to carry out the analysis of these key metabolites combined with corresponding pathways, which served as a guide for enhancement production of secondary metabolites. Furthermore, rational exogenous feeding strategies were proposed according to the results of comparative metabolic profiling analysis towards improvement of betulin production.

## 2. Results

### 2.1. SNP Induces Betulin Accumulation

**Figure 1:**
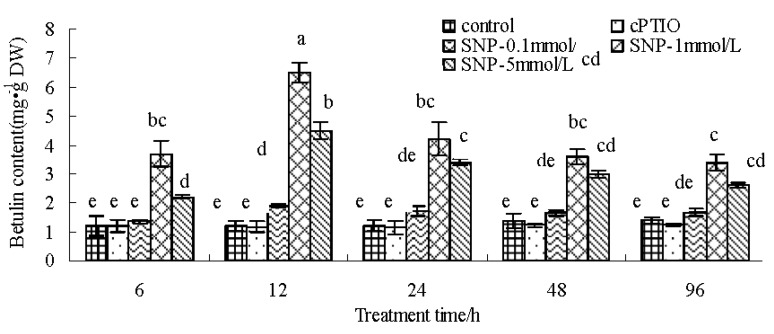
Effect of SNP on betulin production in birch suspension cells. The letter explained the difference of data from statistical level. (*p* < 0.05, Tukey’s test).

Figure 1 shows that the betulin production of the birch suspension cells induced by SNP was time- and dose-dependent. Betulin production increased relatively after SNP treatment, and the maximum betulin content in suspension cells of birch was 6.51 mg/g DW at 12 h after 1 mM SNP treatment, approximately five times that seen in the control. However, the betulin production under cPTIO treatment was similar to that of control.

### 2.2. Identification and Analysis of Key Metabolites and Pathways under SNP Treatment

**Figure 2:**
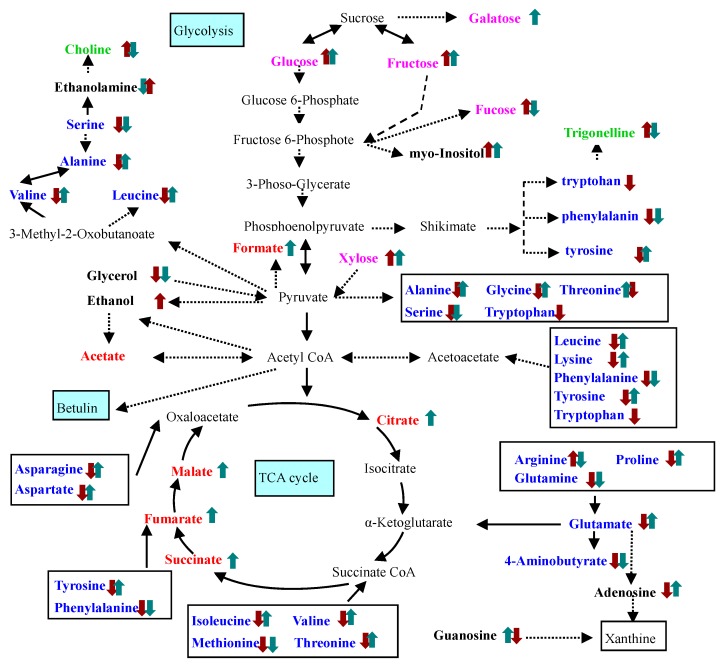
Proposed metabolic pathway alteration in suspension cells of birch induced by NO treatment. Metabolites identified by NMR spectroscopy are depicted in bold (blue amino acids and the derivatives; red Organic acids; pink sugars; green alkaloids; black other metabolites). 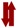 cPTIO treatment, 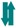 SNP treatment. Dash arrows skip one or several steps.

**Figure A1:**
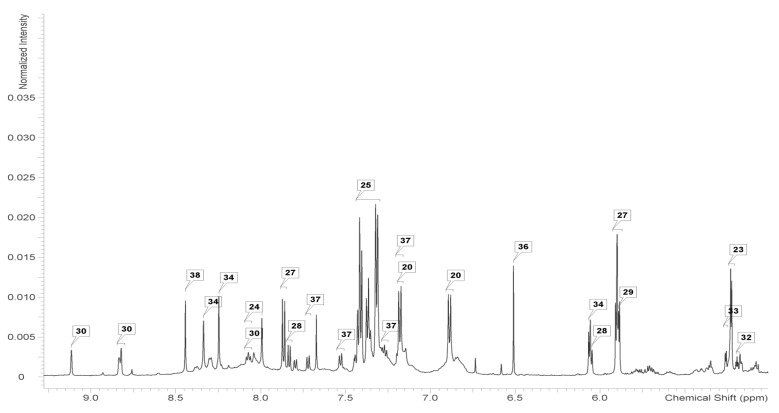
The figure of NMR.

In order to shed light on the reasons for the different betulin production capabilities observed under SNP treatment, intracellular metabolites alterations caused by different SNP treatments were analyzed by ^1^H-NMR (Figure A1). In total, 41 metabolites including 19 amino acids, six organic acids, five sugars, two alkaloids, two purine nucleosides, and seven other metabolites were detected in suspension cells of birch after SNP treatment or cPTIO treatment (Figure 2 and Appendix Table A1). These compounds, involved in the glycolysis pathway, tricarboxylic acid (TCA) cycle, amino acids metabolism and alkaloids metabolism (Figure 2), effectively reflected intracellular metabolic status.

Among them, SNP increased the content of 13 amino acids (except for serine, phenylalanine, arginine, glutamine, 4-aminobutyrate and methionine), six organic acids and two purine nucleosides and five sugars (except for fucose), reduced the content of two alkaloids; cPTIO treatment reduced the content of 18 amino acids (except for arginine) and two purine nucleosides, had no effect on the content of six organic acids, increased the content of five sugars (except for galatose) and two alkaloids (Figure 2 and Table A1).

**Figure 3:**
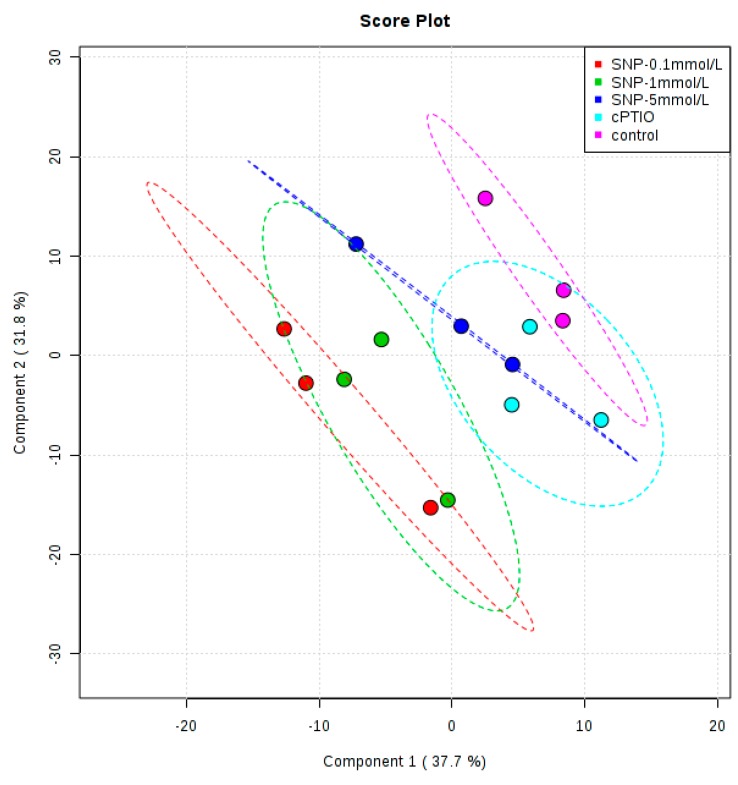
Scores plots of PLS-DA analysis showing the differences between the control and NO treatment.

To examine the metabolic changes in birch cell suspension in response to SNP treatment or cPTIO treatment and the significance of the contributions of the various metabolites to these alterations, the data were analyzed by a supervised chemometric method, partial least squares discriminant analysis (PLS-DA). A permutation (Figure S1) test with 1000 permutations was performed to establish the statistical significance of the models. Figure S1 shows the permutation plots, revealing that the original PLS-DA models were valid. The PLS-DA score plot showed that the control was separated from all SNP treatment concentrations (Figure 3). The good separation on the PLS profile revealed the metabolic difference caused by SNP treatment. However, the metabolic profiling of cPTIO treatment was overlaped with control and 5 mmol/L SNP treatment. This indicated that cPTIO treatment had some similar metabolites with that of control.

**Figure 4:**
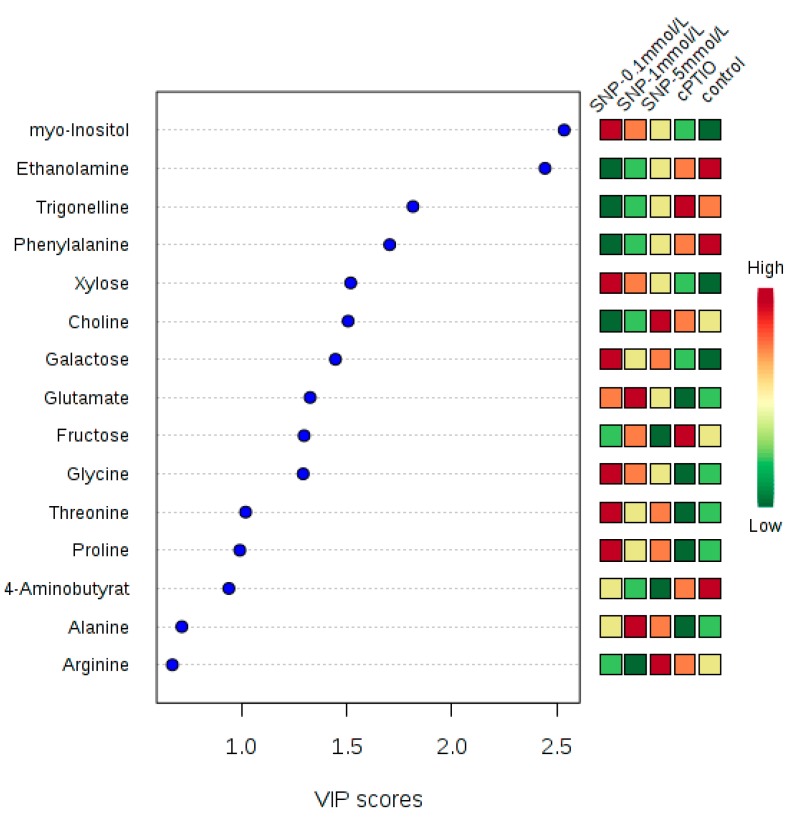
Identified 10 metabolites which had VIP value greater than 1 after NO treatment.

Next, the VIP coefficient reflected the contribution of each identified metabolite to samples class separation (Figure 4). The higher VIP value meant that the metabolite had a larger contribution. Generally, a metabolite with a VIP greater than 1 illustrated a significant contribution to the separation of sample groups within PLS model. In this work, a total of 10 metabolites which had VIP value greater than 1 were identified. They were myo-inositol, ethanolamine, trigonelline, phenylalanine, xylose, choline, galactose, glutamate, fructose and glycine. These metabolites (VIP > 1) were mainly involved in the central carbon metabolism and amino acid metabolism. 

### 2.3. Enhancing the Betulin Production by Combinatorial Feeding Strategy

**Figure 5:**
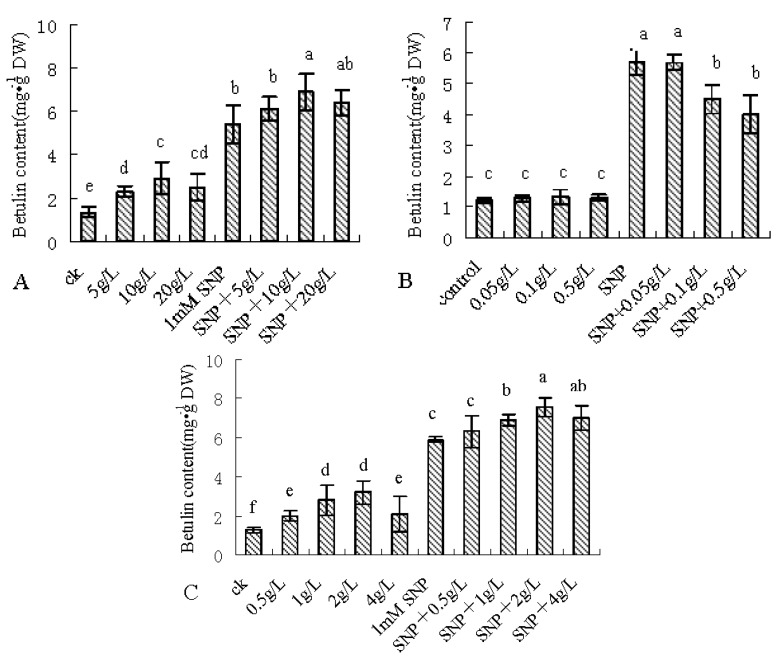
Effects of SNP combined with fructose, myo-inositol or phenylalanine on betulin content in birch suspension cells. 8-day-old suspension cells were treated with 1 mmol/L SNP combined with 3 g/L, 6 g/L, 10 g/L and 20 g/L fructose (**A**); 0.05 g/L, 0.5 g/L, 5 g/L myo-inositol (**B**); 0.5 g/L, 1 g/L, 2 g/L and 4 g/L phenylalanine (**C**); and harvested 12h after treatment. The letter explained the difference of data from statistical level. (*p* < 0.05, Tukey’s test).

To validate the contribution of 10 metabolites which had VIP value greater than 1 on betulin production. Correlation analysis was used to evaluate the association between the content of 10 metabolites and betulin under 1 mmol/L SNP. The results showed that SNP treatment changed the correlation between the content of 10 metabolites and betulin (Table A2). Among them, SNP treatment improved the correlation between myo-inositol, fructose and betulin, respectively. But the correlation between phenylalanine and betulin was reduced from 0.994 (in control plants) to −0.418 (SNP-treated plants), so the above three metabolites were chosen to feed birch suspension cell culture under SNP treatment. Exogenous feeding results showed that fructose and SNP treatment or phenylalanine and SNP treatment further enhanced betulin production, the maximum increasing rate was 28.8% comparing with SNP treatment, but myo-inositol and SNP treatment had the opposite result (Figure 5).

## 3. Discussion

In a previous report, SNP treatment increased the gene expression of key enzymes in the triterpenoid biosynthesis pathway in suspension cells of birch. For example, 3-hydroxy-3-methylglutaryl coenzyme A reductase (HMGR), the formation of methylerythritol 4-phosphate is catalyzed by deoxyxylulose phosphate isomerase (DXR), 2,3-oxidosqualene can be synthesized in the presence of squalene epoxidase (SQS) and then formation of oleanolic acid and betulin catalyzed by β-amyrenol and lupeol synthase, respectively [8,9,14]. The main pentacyclic triterpenoids in birch were betulin, betulinic acid and oleanolic acid [15]. On this study, we verified that SNP treatment enhanced the betulin production. This result was consistent with the above result at transcription level.

In the study betulin production associated metabolites and metabolic remobilization under NO treatment, profiling of metabolites, including sugars, amino acids, organic acids and secondary metabolites (in total, approximately 41 annotated metabolites), was performed using samples obtained from suspension cells of birch. The results showed that the response to NO treatment was much more than the simple induction of production of triterpenoid.

Among the 41 annotated metabolites, only the content of glucose and fructose in carbohydrate metabolism were all enhanced at SNP treatment and cPTIO treatment, and the result of SNP treatment was consistent with that of transcription level. The increased glucose and fructose at SNP treatment and cPTIO treatment was, at a first glance, difficult to interpret. Further statistical analysis showed that VIP value of fructose was greater than 1, and its correlation with betulin content was from −0.866 under cPTIO treatment to 0.530 under SNP treatment. These indicated that fructose could be used as the identified metabolite to differentiate different NO treatments, and had great contribution for betulin production. Combinatorial feeding strategies under SNP and fructose treatment further enhanced betulin production. The above results suggested that fructose as a portion of the diverted carbon was shifted toward betulin production.

The response of 13 amino acids (except for serine, phenylalanine, arginine, glutamine, 4-aminobutyrate and methionine), six organic acids, two purine nucleosides and two alkaloids to different NO treatments were opposite. SNP treatment increased the content of 13 amino acids, six organic acids and two purine nucleosides, and reduced the content of two alkaloids. These results indicated NO treatment altered shikimate, amino acids metabolism, alkaloids metabolism and organic acids metabolism in TCA. The above change in metabolism may change the carbon, nitrogen and energy metabolism, in turn, increase of betulin production.

The shikimate pathway produces the three proteinogenic aromatic amino acids, phenylalanine, tyrosine and tryptophan, which are, in addition to several intermediates of the shikimate pathway, intermediates in the biosynthesis of numerous aromatic natural products in higher plants [16]. Trigonelline is one of their downstream products. In previous resport, UV-B or salt treatment enhanced the trigonelline production, and increased the phenylalanine ammonia-lyase (PAL) activity [17,18], but our study showed that SNP treatment decreased the content of phenylalanine, tyrosine and trigonelline, and had no effect on tryptophan. The other alkaloid choline obtained similar result derived from the serine under SNP treatment. NO was the product of UV-B or salt treatment, and had different effect on trigonelline production. The reason may be a result of using different kinds of samples.

In the secondary metabolites biosynthesis of plant cell cultures, some precursor feeding at suitable concentrations with optimal exposure time can elevate the synthesis of secondary metabolites, but on the other hand, excess precursor concentration with improper exposure time may cause feedback inhibition of metabolite pathways [19]. Determination of appropriate precursors and their concentration in precursor-feeding is essential to achieve higher production of secondary metabolites [20,21]. Our exogenous feeding results based on comparative metabolic profiling showed that fructose or phenylalanine treatment further enhanced the production of betulin under SNP treatment, but myo-inositol had the opposite result. Myo-inositol is a sugar-like carbohydrate produced by most plants, and is important for phosphate storage, cell wall biosynthesis, the production of stress related molecules, cell-to-cell communication, storage and transport of plant hormones [22]. Its detailed reason for above result in betulin production needs further study.

There also are notable gaps in our understanding the relationship between plant primary metabolism and secondary metabolism under NO treatment. Usually the major secondary metabolism classes produced by plants can be divided into three main groups: firstly, phenolic compounds; secondly, terpenoids/isoprenoids; and thirdly, nitrogen or sulfur containing compounds such as the alkaloids and glucosinolates, respectively. The three major classes of secondary metabolisms are produced from pathways of different primary metabolisms, including glycolysis, the TCA cycle, aliphatic amino acids, pentose phosphate pathway, shikimate pathway and notably the aromatic amino acids (AAAs) [23]. However, ^1^H-NMR analysis could not examine whole metabolites in living cells in our study. Therefore, more qualitative and quantitative information of metabolome are needed to comprehensive understand cellular differentiation pathway. Also metabonomics integration with transcriptome and proteome will give new insights towards the accumulation of secondary metabolism under NO treatment.

## 4. Materials and Methods

### 4.1. Plant Cell Culture

The cell line used in the study was developed from the axillary buds of a 30-year-old *B. platyphylla* Suk. Suspension cultures were established from this cell line and cultivated on optimized Nagata–Takebe medium supplemented with 0.1 mg/L 6-benzyladenine, 0.01 mg/L thidiazuron, and 20 g/L sucrose (Table A3). The medium was adjusted to pH 5.6 and then sterilized by autoclaving at 121 °C for 20 min. The suspension culture was maintained in 250 mL Erlenmeyer flasks, with a liquid volume of 100 mL in each flask, and inoculated with 4.0 g fresh weight of 8-day-old cell suspension cultures. The Erlenmeyer flasks were incubated on a rotary shaker (110 rpm) at 25 °C. Illumination was regulated to give 14/10 h “photoperiod” (photophase 06: 00 h–20: 00 h) provided by fluorescent tubes (mixture of Osram fluora and Osram daylight types) with a photon flux density (2000 lux).

### 4.2. Chemical Reagents and Treatment

Suspension cells were fed with 150 μmol/L 2-(4-carboxyphenyl)-4,4,5,5-tetramethyl-imidazoline-1-oxyl-3-oxide (cPTIO), an NO-specific scavenger; 0.1 mmol/L, 1 mmol/L and 5 mmol/L sodium nitroprusside (SNP) treatment, an NO donor, and harvested during 6 h–96 h after treatment (Figure 1); Suspension cells were treated with 150 μmol/L cPTIO, 0.1 mmol/L, 1 mmol/L and 5 mmol/L SNP treatment, and harvested at 12 h after treatment (Figure 2, Figure 3 and Figure 4); suspension cells were treated with 1 mmol/L SNP combined with 3 g/L, 6 g/L, 10 g/L and 20 g/L fructose (Figure 5A), 0.05 g/L, 0.5 g/L, 5 g/L myo-inositol(Figure 5B) and 0.5 g/L, 1 g/L, 2 g/L and 4 g/L phenylalanine (Figure 5C), and harvested 12 h after treatment. Among them, SNP and cPTIO were purchased from Sigma Corporation (St. Louis, MO, USA), other chemicals were purchased Beijing Huagong Biotechnology Institute (Beijing, China). For chemical treatments, 8-day-old suspension cells were treated with the above chemicals. The controls were treated with the same volume of distilled water. The concentrations of chemical reagents were selected based on our preliminary experiments. Values are mean ± SE (*n* = 3).

### 4.3. Betulin Estimation

Betulin was extracted from birch suspension cells using a procedure similar to that reported by Fan et al. using ethanol as the solvent [8]. The solvent was removed by evaporation under vacuum, and the solid residue was re-dissolved in ethanol. The betulin content in the ethanol sample solution was analyzed by reversed-phase HPLC with ultraviolet detection at 210 nm. We used a Waters C_18_ HPLC column (250 mm × 4.6 mm with 5 μm packing). All sample solutions for HPLC analysis were filtered through a 0.45 μm membrane filter before injection. The mobile phase consisted of 80:20 (*v/v*) acetonitrile: water. The flow rate was 1 mL/min. The betulin standard was obtained from Sigma-Aldrich Chemical Corporation (St. Louis, MO, USA).

### 4.4. ^1^H-NMR Measurements

For ^1^H-NMR analysis, 50 mg freeze-dried suspension cells were transferred into a centrifuge tube. 1800 μL extraction solution (water:methanol:chloroform = 1:1:1, *v/v*) were added to the test tubes, vortexed for 2 min and sonicated for 1min, and then the materials were centrifuged at 500× *g* for 10 min. The supernatants were transferred separately into a 50 mL round bottomed vial to dry in vacuum freeze dryer. 600 μL phosphate buffer solution (0.1 M, pH 7.0) and 50 μL Anachro certified DSS standard solution were added to the above dried samples. The samples were centrifuged at 500× *g* for 15 min and the supernatants were transferred into 5-mm NMR tubes for NMR measurements.

### 4.5. Data Processing and Statistical Analysis

All of the ^1^H-NMR spectra were collected at 25 °C on a 600 MHz AV III spectrometer (Bruker Corporation, Switzerland) equipped with an inverse cryoprobe. CD3OD was used as the internal lock. Each ^1^H-NMR spectrum consisted of 128 scans complying with the parameters used by Yang et al. [24]. ^1^H-NMR spectra were acquired using the first transient of the Bruker “noesygppr1d.comp” sequence. All free induction decay (FID) signals were input into the Chenomx NMR Suite Professional software (version 7.7, Chenomx, Edmonton, AB, Canada). Then, the automatically adjusted phase was corrected to the baseline. The DSS-*d*_6_ groups were used as internal standard references while determining the chemical shift (set to 0 ppm) in all spectra; the convolution was reversed and adjusted based on the chemical shift index (CSI) and peak shape in the spectrum. According to the related information in the ^1^H-NMR spectra (chemical shift, peak shape, half band width, and coupling split classification), we used the DSS-*d*_6_ concentration and the peak area as standards. Finally, the Chenomx NMR suite software and an internal database (a standard Chenomx metabolite database) were used to perform qualitative and quantitative analyses of the NMR spectra.

Partial least square-discriminant analysis (PLS-DA) was performed with the SIMCA-P software (Umetrics, Umeå, Sweden). Furthermore, Variable Influence on Projection (VIP) values of PLS-DA models were used to extract the most influential metabolites contributing to group separation, and only metabolites with VIP scores > 1 were considered significant, and they were partially responsible for the differences between SNP treatment and cPTIO treatment and control subjects. Statistically significant metabolites were further determined by Student paired *t*-test (*p* < 0.05). All experiments were conducted in triplicate. Data (mean ± standard error) were statistically analyzed using SPSS version 15.0 (IBM, Armonk, NY, USA). We used *t*-tests for simple comparisons between each treatment and its control, and Tukey’s tests for multiple comparisons among means. The assumptions of analysis of variance were considered to be statistically significant at *p* < 0.05.

## Supplementary Materials

Supplementary materials are available online. Figure S1: Permutation test figure.

## Appendix A

**Table A1:** Metabolites alterations caused by different NO treatments analyzed by NMR.

Metabolite	Control	cPTIO	SNP 0.1 mmol/L	SNP 1 mmol/L	SNP 5 mmol/L
**Sugars**					
Fructose	3.23 ± 0.56a	4.14 ± 0.54a	3.28 ± 0.77a	3.90 ± 0.61a	2.76 ± 0.45a
Fucose	0.10 ± 0.01a	0.11 ± 0.04a	0.09 ± 0.01a	0.08 ± 0.01a	0.09 ± 0.02a
Galactose	0.24 ± 0.07a	0.24 ± 0.06a	0.41 ± 0.10ab	0.35 ± 0.11b	0.32 ± 0.06ab
Glucose	1.50 ± 0.11b	1.89 ± 0.38b	1.80 ± 0.24b	1.87 ± 0.15b	1.04 ± 0.12a
Xylose	0.16 ± 0.01a	0.19 ± 0.05a	0.36 ± 0.07ab	0.25 ± 0.04b	0.18 ± 0.06a
**Organic Acids**					
Acetate	0.05 ± 0.00a	0.05 ± 0.01a	0.03 ± 0.02a	0.05 ± 0.01a	0.06 ± 0.01a
Citrate	0.07 ± 0.01a	0.07 ± 0.01a	0.06 ± 0.02a	0.08 ± 0.01a	0.06 ± 0.02a
Formate	0.21 ± 0.03abc	0.21 ± 0.0ab	0.15 ± 0.04c	0.29 ± 0.06a	0.28 ± 0.05bc
Fumarate	0.13 ± 0.04a	0.13 ± 0.03a	0.12 ± 0.03a	0.16 ± 0.05a	0.10 ± 0.01a
Malate	5.62 ± 0.33a	5.62 ± 0.54a	6.42 ± 0.13a	6.45 ± 0.71a	4.95 ± 0.90a
Succinate	0.34 ± 0.11a	0.34 ± 0.03a	0.37 ± 0.10a	0.49 ± 0.09a	0.32 ± 0.07a
**Amino Acids and the Derivatives**					
4-Aminobutyrate	0.17 ± 0.05c	0.13 ± 0.01bc	0.11 ± 0.02abc	0.11 ± 0.02ab	0.06 ± 0.01a
Alanine	0.88 ± 0.07a	0.78 ± 0.08a	0.94 ± 0.10a	1.13 ± 0.20a	0.88 ± 0.09a
Arginine	0.21 ± 0.04ab	0.22 ± 0.03b	0.21 ± 0.05a	0.13 ± 0.01ab	0.20 ± 0.05ab
Asparagine	0.16 ± 0.04a	0.15 ± 0.01a	0.18 ± 0.05a	0.21 ± 0.04a	0.21 ± 0.03a
Aspartate	0.18 ± 0.02a	0.17 ± 0.02a	0.24 ± 0.08a	0.19 ± 0.06a	0.24 ± 0.05a
Glutamate	0.48 ± 0.07ab	0.37 ± 0.08a	0.61 ± 0.11b	0.68 ± 0.10ab	0.50 ± 0.09ab
Glutamine	0.19 ± 0.05a	0.17 ± 0.04a	0.18 ± 0.06a	0.17 ± 0.02a	0.18 ± 0.03a
Glycine	0.27 ± 0.06a	0.20 ± 0.03a	0.40 ± 0.12a	0.38 ± 0.04a	0.28 ± 0.09a
Isoleucine	0.42 ± 0.07a	0.35 ± 0.06a	0.41 ± 0.09a	0.43 ± 0.08a	0.35 ± 0.08a
Leucine	0.67 ± 0.09a	0.53 ± 0.10a	0.71 ± 0.13a	0.75 ± 0.12a	0.66 ± 0.12a
Lysine	0.26 ± 0.09a	0.20 ± 0.04a	0.30 ± 0.05a	0.25 ± 0.07a	0.21 ± 0.05a
Methionine	0.21 ± 0.05a	0.20 ± 0.03a	0.19 ± 0.03a	0.20 ± 0.02a	0.15 ± 0.01a
Phenylalanine	0.69 ± 0.03a	0.60 ± 0.12a	0.52 ± 0.10a	0.65 ± 0.07a	0.52 ± 0.06a
Proline	0.18 ± 0.02a	0.14 ± 0.05a	0.29 ± 0.03a	0.23 ± 0.07a	0.22 ± 0.06a
Serine	0.35 ± 0.04a	0.26 ± 0.02a	0.36 ± 0.03a	0.34 ± 0.06a	0.29 ± 0.04a
Threonine	0.33 ± 0.07a	0.30 ± 0.04a	0.44 ± 0.08a	0.44 ± 0.01a	0.36 ± 0.08a
Tryptophan	0.11 ± 0.01a	0.10 ± 0.01a	0.09 ± 0.01a	0.11 ± 0.03a	0.10 ± 0.01a
Tyrosine	0.19 ± 0.02a	0.18 ± 0.03a	0.23 ± 0.08a	0.25 ± 0.06a	0.21 ± 0.05a
Valine	0.49 ± 0.04a	0.43 ± 0.08a	0.58 ± 0.02a	0.58 ± 0.07a	0.44 ± 0.06a
**Alkaloids**					
Choline	0.54 ± 0.01ab	0.61 ± 0.05b	0.45 ± 0.08ab	0.51 ± 0.02a	0.54 ± 0.04ab
Trigonelline	0.22 ± 0.02b	0.26 ± 0.08b	0.09 ± 0.02a	0.13 ± 0.03a	0.17 ± 0.02ab
**Other Metabolites**					
Ethanolamine	0.85 ± 0.09bc	0.88 ± 0.03c	0.60 ± 0.08abc	0.68 ± 0.04a	0.64 ± 0.11ab
Uridine	0.25 ± 0.04a	0.30 ± 0.03a	0.24 ± 0.01a	0.30 ± 0.03a	0.22 ± 0.02a
Adenosine	0.13 ± 0.02a	0.12 ± 0.02a	0.10 ± 0.01a	0.14 ± 0.03a	0.13 ± 0.03a
Ethanol	0.02 ± 0.00a	0.03 ± 0.00a	0.03 ± 0.00a	0.02 ± 0.00a	0.03 ± 0.00a
myo-Inositol	0.66 ± 0.08a	0.88 ± 0.14a	1.12 ± 0.15b	1.19 ± 0.06b	0.84 ± 0.09a
Glycerol	0.40 ± 0.05a	0.34 ± 0.03a	0.40 ± 0.01a	0.37 ± 0.04a	0.38 ± 0.08a
Cytidine	0.12 ± 0.04a	0.13 ± 0.02a	0.10 ± 0.00a	0.13 ± 0.01a	0.13 ± 0.02a
Guanosine	0.14 ± 0.02a	0.12 ± 0.03a	0.12 ± 0.01a	0.16 ± 0.03a	0.15 ± 0.02a
Methanol	0.38 ± 0.01a	0.26 ± 0.04a	0.20 ± 0.03a	0.31 ± 0.01a	0.24 ± 0.04a

Note: The letter explained the difference of data from statistical level. (*p* < 0.05, Tukey’s test).

**Table A2:** The correlation between the content of 12 metabolites which had VIP value greater than 1 and betulin content.

Metabolites	Control	cPTIO	SNP(1 mmol/L^−1^)
Myo−inositol	0.015	0.992	0.443
Ethanolamine	0.249	−0.347	−0.697
Trigonelline	−0.545	−0.696	−0.078
Phenylalanine	0.994	0.542	−0.418
Xylose	0.998	−0.907	−0.681
Choline	0.719	−0.243	0.217
Galactose	0.600	−0.620	−0.917
Glutamate	0.277	0.371	−0.869
Fructose	−0.246	−0.866	0.530
Glycine	−0.319	0.955	−0.748

**Table A3:** NT culture medium component (mg/L).

Component	NT
MgSO4·7H_2_O	1233
CaC1_2_·2H_2_O	220
KNO_3_	950
NH_4_NO_3_	825
KH_2_PO_4_	680
FeSO_4_·7H_2_O	27.8
Na_2_-EDTA	37.3
MnSO_4_·4H_2_O	22.3
KI	0.83
CoC1_2_·6H_2_O	0.03
ZnSO_4_·7H_2_O	8.6
CuSO_4_·5H_2_O	0.25
H_3_BO_3_	6.2
Na_2_MoO_4_·2H_2_O	0.25
Inositol	100
VB_1_	1
Glycine	3
