# Supplementary figures and images for: Exogenous Feeding of Fructose and Phenylalanine Further Improves Betulin Production in Suspended Betula platyphylla Cells under Nitric Oxide Treatment
Source: Molecules. 2017 Jun 30;22(7):1035. doi: 10.3390/molecules22071035 (PMC6152328; doi:10.3390/molecules22071035)

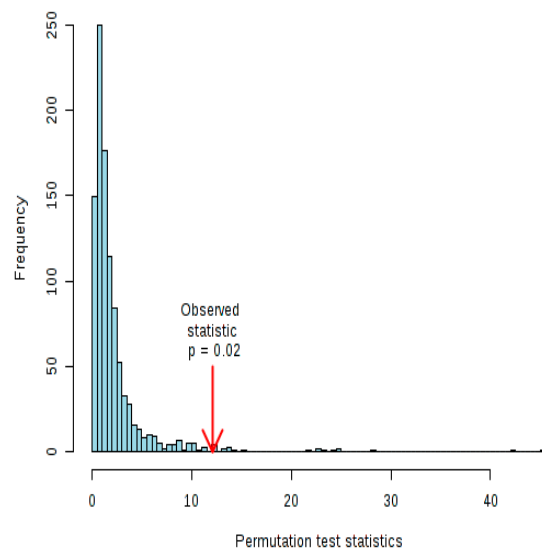

Fig. S1 Permutation test figure.

Supplement: Supplementary file 1 [file molecules-22-01035-s001.pdf]
